# Peripheral mRNA Expression and Prognostic Significance of Emotional Stress Biomarkers in Metastatic Breast Cancer Patients

**DOI:** 10.3390/ijms232214097

**Published:** 2022-11-15

**Authors:** Tahreem Fiaz, Muhammad Shahid Nadeem, Obaid Afzal, Abdulmalik S. A. Altamimi, Sami I. Alzarea, Waleed Hassan Almalki, Hafsa Ahmed Khan, Iahtisham-Ul-Haq, Sharoon Hanook, Imran Kazmi, Muhammad Mustafa

**Affiliations:** 1 Kauser Abdulla Malik School of Life Sciences, Forman Christian College (A Chartered University), Lahore 54600, Pakistan; 2 Department of Biochemistry, Faculty of Science, King Abdulaziz University, Jeddah 21589, Saudi Arabia; 3 Department of Pharmaceutical Chemistry, College of Pharmacy, Prince Sattam Bin Abdulaziz University, Al-Kharj 11942, Saudi Arabia; 4 Department of Pharmacology, College of Pharmacy, Jouf University, Sakaka 72341, Saudi Arabia; 5 Department of Pharmacology, College of Pharmacy, Umm Al-Qura University, Makkah 21955, Saudi Arabia

**Keywords:** emotional stress, HPA-axis, stress-related genes, breast cancer, distress, prognosis

## Abstract

Emotional stress is believed to be associated with increased tumor progression. Stress-induced epigenetic modifications can contribute to the severity of disease and poor prognosis in cancer patients. The current study aimed to investigate the expression profiles along with the prognostic significance of psychological stress-related genes in metastatic breast cancer patients, to rationalize the molecular link between emotional stress and cancer progression. We profiled the expression of selected stress-associated genes (*5-HTT, NR3C1, OXTR,* and *FKBP5*) in breast cancer including the stress evaluation of all participants using the Questionnaire on Distress in Cancer Patients–short form (QSC-R10). A survival database, the Kaplan–Meier Plotter, was used to explore the prognostic significance of these genes in breast cancer. Our results showed relatively low expressions of *5-HTT* (*p* = 0.02) and *OXTR* (*p* = 0.0387) in metastatic breast cancer patients as compared to the non-metastatic group of patients. The expression of *NR3C1* was low in tumor grade III as compared to grade II (*p* = 0.04). Additionally, the expression of *NR3C1* was significantly higher in patients with positive estrogen receptor status. However, no significant difference was found regarding *FKBP5* expression in breast cancer. The results suggest a potential implication of these genes in breast cancer pathology and prognosis.

## 1. Introduction

Psychological stress is a non-tangible external element that has the ability to affect the physiology of organisms [1]. The effects of stress were first observed by Hans Selye, an Austrian endocrinologist who acclaimed it as a syndrome that appears in response to diverse nonspecific stimuli such as changes in body temperature, surgical injury, hyperphysical activity, and drug administration [2].

Chronic stress can challenge the homeostasis of organisms by altering a wide range of normal physiological processes [3]. Behavioral studies have found that constant emotional distress can alter the psychological in addition to physiological characteristics of cancer patients, especially those suffering from breast cancer [4,5] Conventional cancer therapies also compromise the life quality of cancer patients [6], and can further trigger factors like chronic depression, anxiety, and insomnia [7].

Impaired activity of the stress system can enhance the severity of many pathological conditions [8,9,10]. It has been suggested that emotional stress can enhance the invasiveness and proliferation of tumors in several cancers [11,12,13]. Persistent stress can over-activate the Hypothalamus Pituitary adrenal axis (HPA axis) [14], impairing the circadian rhythms [15] and deregulating immune response [16], which can ultimately contribute to the severity of several cancers.

Living in a developing country, breast cancer is one of the most common and emotionally challenging diseases. It has been identified that not only a genetic predisposition but epigenetic modifications also play a significant role in cancer [17]. Emotional stress might be one of the factors that can accelerate metastasis through epigenetic means.

Our research aimed to investigate the molecular premises of emotional stress-related disease burden in cancer patients, with a focused interest to study the transcriptional profiles of stress-associated genes in breast cancer. We have identified four genes (*NR3C1, 5-HTT, OXTR*, and *FKBP5*) based on previously reported genes that were found hyper-methylated in specific psychological abnormalities [18]. We have studied whether the mRNA expression of stress-associated genes is related with the clinical characteristics of breast cancer patients. Investigating the role of stress-related factors in cancer metastasis can help in identifying the potential involvement of stress in cancer proliferation and providing future insights on stress-related signaling cascades associated with breast cancer.

## 2. Materials and Methods

### 2.1. Study Design and Participants

We conducted our research at the KAM School of Life Sciences, Forman Christian College (A Chartered University), Lahore, Pakistan. The project was authorized by the Institutional Review Board (IRB) and Ethical Review Committee (ERC) at the Forman Christian College (A Chartered University), Lahore, Pakistan. A total of 32 breast cancer patients receiving chemotherapy were enrolled in the study. All patients participated voluntarily and signed a consent form prior to their enrollment in the study. The blood samples were withdrawn by a trained medical officer. The privacy and anonymity of all participants have been respected to prevent harm to vulnerable populations and to ensure the integrity of our research. The demographic representation of all subjects is mentioned in Table 1.

### 2.2. Psychometric Stress Evaluation

The stress analysis of breast cancer patients was performed using a Questionnaire on Distress in Cancer Patients–short form (QSC-R10). QSC-R10 is a short form consisting of ten screening items used to evaluate cancer-related psychological stress in cancer patients [19]. It is essential to assess whether a patient needs psycho-oncological support as it can reflect the patients’ subjective experiences of their disease. The ten elements of QSC-R10 are shown in Figure S1. The validity of the questionnaire has already been determined by professionals to measure cancer-related stress in patients [16].

### 2.3. Blood Sampling and Data Collection

The blood samples of 32 breast cancer patients were collected. For each collection, 5 mL of blood was drawn from a peripheral venous catheter in EDTA-coated vials. The invasive procedures for the sample collection were performed by trained medical staff at the hospital to avoid any injury. The samples were stored and transferred to the institutional research laboratory. The clinical presentation and medical history of each patient were obtained using a data collection form.

### 2.4. Quantitative RT-PCR for Gene Expression Profiling

#### 2.4.1. RNA Extraction and cDNA Synthesis

Total RNA was extracted from fresh blood samples using TRIzol™ reagent (Invitrogen). The concentration and ratio of absorbance (260/280) of RNA were measured using a Nanodrop machine. The cDNA synthesis was performed by using a Thermo Scientific Revert Aid First Strand cDNA Synthesis Kit (#K1621). First-strand cDNA synthesized with this system can be directly used as a template in PCR or real-time PCR. The normalization of cDNA samples was performed using *B-ACTIN* as a reference gene [20].

#### 2.4.2. Primers for RT-PCR

Primers were designed for the specific genes (NR3C1, 5-HTT, OXTR, and FKBP5) to observe their expression analysis. The mRNA sequence of these genes was recruited from the National Center of Biotechnology Information (NCBI). After specific primers were designed, and oligo synthesis was performed by Eurofins Genomics. Confirmation of product size and annealing temperatures was performed by in silico PCR using a USCS genome browser. The primer sequences of the selected genes are given in Table 2.

#### 2.4.3. Quantification of Gene Expression

The genes were expression-quantified using semi-quantitative digital analysis [20] with the help of ImageJ (National Institutes of Health, Bathesda, Maryland, USA). The software helps to measure the band intensity, its area, and pixels. The band intensity of each sample PCR product for the reference gene *(B-ACTIN*) as well as our specific genes for analysis was calculated. Relative gene expression values were determined by calculating the ratio between the values of our specific genes and the housekeeping gene (*B-ACTIN)*.

### 2.5. Survival Analysis of Breast Cancer Patients Using a Kaplan–Meier Plotter

The survival plots were recruited from a Kaplan–Meier plotter “https://kmplot.com/analysis/ (accessed on 26 July 2021)”, a prognostic database that is used to analyze the prognostic effect of a certain gene on breast cancer [21]. The analysis was based on overall survival (OS), recurrence-free survival (RFS), distant-free metastatic survival (DFMS), and post-progression survival (PPS) for our genes. The Affymetrix IDs for data sets are: *NR3C1* (201865_x_at), *5-HTT* (207519_at), *OXTR* (206825_at), and *FKBP5* (204560_at). The hazard ratio with 95% confidence intervals and log-rank *p* values were noted.

### 2.6. Statistical Analysis

Statistical analysis was performed using the GraphPad Prism 9.3.0 version. Student’s *t*-test was used to test the significance among the different variables. Quantitative results were expressed as means ± SEM. Differences were considered statistically significant at *p* ≤ 0.05. All Kaplan–Meier survival plots were reported with a *p*-value obtained using a log-rank test by the Kaplan–Meier plotter database.

## 3. Results

### 3.1. Distress Screening Using QSC R-10

Stress scores of the patients were calculated using QSC-R10 as described in the methods. The average stress score of all patients (n = 32) was 3.16, indicating the moderate presence of cancer-related stressors. Patients were further divided into subgroups based on their age, mastectomy, illness duration, and their metastatic status to find any significant difference in average stress scores among these variables. The statistical significance was measured using Student’s *t*-test for “mastectomy” and “metastasis’ and one-way ANOVA was conducted for “Age” and “Illness duration”. The results showed significantly high stress scores among the patients of ages ranging from 35–45 (Table 3). Additionally, significantly high average stress scores were found in the patients who had gone through a mastectomy and those with illness durations of less than 6 months (Table 3). A post hoc comparison of means was also performed among multiple variable groups (illness duration and age). The groups with different alphabetical letters show statistically significant results. However, we found no significance in terms of patient metastatic status.

### 3.2. Expression and Prognostic Significance of Stress-Related Genes in Breast Cancer

The peripheral expression of *5-HTT, NR3C1, OXTR*, and *FKBP5* was measured as described in the methods. We found that these genes were differentially expressed in cancer patients depending upon the cancer stage and tumor grade. Moreover, the prognostic association of these genes with survival outcomes was found out by using the Kaplan–Meier plotter. The KM survival plots predict a range of survival types in different types of cancer: overall survival (OS), Recurrence Free Survival (RFS), Post-Progression survival (PPS), and Distant Free Metastatic survival (DFMS). The association of survival probabilities with the expression of these genes is shown in Table 4.

#### 3.2.1. Serotonin Transporter (*5-HTT*) in Breast Cancer

**Figure 1:**
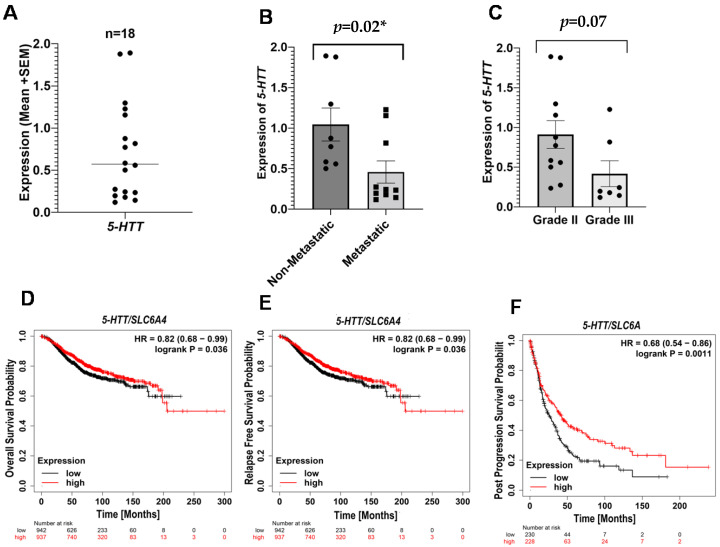
Expression of *5-HTT* and survival outcomes in breast cancer patients. Peripheral expression of *5-HTT* across all breast cancer patients (**A**). Difference in the expression of 5-HTT between non-metastatic and metastatic patients (**B**). Difference in the expression of 5-HTT between grade II and grade III (**C**). Kaplan–Meier survival plots for OS (**D**), RFS (**E**), and PPS (**F**). The results were acquired by Kaplan–Meier Plotter using a log-rank test, and *p* < 0.05 was considered significant. * = significant value.

We observed a significant downregulation in the expression of *5-HTT* (*p* = 0.02 *) in metastatic breast cancer patients compared to the non-metastatic controls (Figure 1B). Additionally, we did not detect any significant difference in the *5-HTT* mRNA levels between grade II and grade III patients (Figure 1C). Moreover, the survival analysis plots for *5-HTT* are also in line with our findings. The probabilities for OS, RFS, and PPS were found to be higher in the patients with a high expression of *5-HTT* (Figure 1D–F). The results suggest that there is an association between low *5-HTT* levels with poor survival and disease progression in breast cancer.

#### 3.2.2. *NR3C1* in Breast Cancer

**Figure 2:**
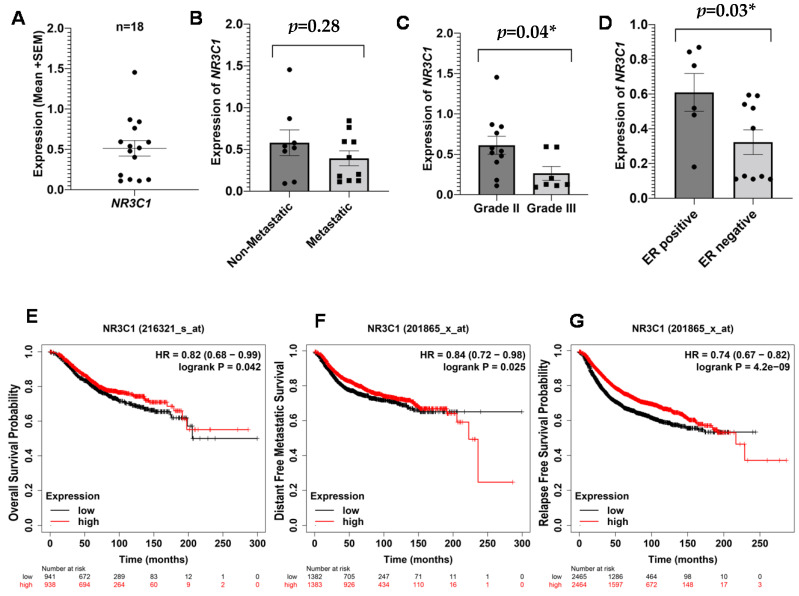
Expression of *NR3C1* and survival outcomes in breast cancer patients. Peripheral expression of *NR3C1* across all breast cancer patients (**A**). Difference in the expression of *NR3C1* between non-metastatic and metastatic patients (**B**). Difference in the expression of *NR3C1* between grade II and grade III (**C**). Difference in the expression of *NR3C1* between ER-positive and ER-negative patients (**D**) Kaplan–Meier survival plots for OS (**E**), DFMS (**F**), and RFS (**G**). The results were acquired by Kaplan–Meier Plotter using a log-rank test, and *p* < 0.05 was considered significant. * = significant value.

*NR3C1* encodes glucocorticoid receptors (GR), which regulate various physiological processes, mainly cellular responses to stress hormones [22]. The expression of *NR3C1* in all breast cancer patients is shown in Figure 2A. We have found a low expression of *NR3C1* in metastatic and grade III patients as compared to non-metastatic and grade II patients, respectively (Figure 2B,C). However, the difference in the expression was only significant for tumor grades (*p* = 0.04). We have also found significantly higher expression of *NR3C1* (*p* = 0.02), in ER-positive patients (Figure 2D), suggesting its role in ER regulation. In addition to gene expression results, Kaplan–Meier analysis confirmed poor probability of OS, RFS, and DMFS (Figure 2E,G). Like *5-HTT*, it is suggested that low expression of *NR3C1* is also associated with poor pathological outcomes in breast cancer.

#### 3.2.3. Oxytocin Receptor (OXTR) in Breast Cancer

**Figure 3:**
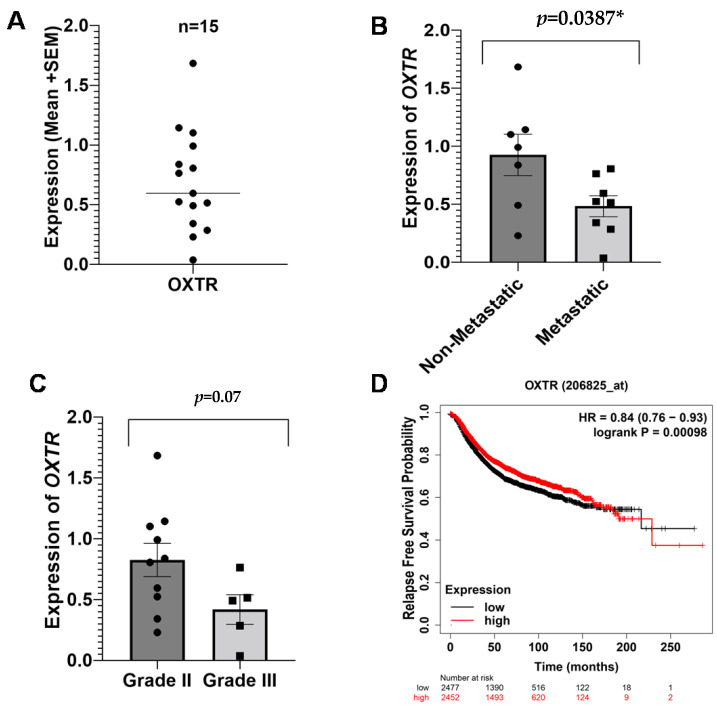
Expression of OXTR and survival outcomes in breast cancer patients. Peripheral expression of OXTR across all breast cancer patients (**A**). Difference in the expression of OXTR between non-metastatic and metastatic patients (**B**). Difference in the expression of OXTR between grade II and grade III (**C**). Kaplan–Meier Survival plots for RFS (**D**). The results were acquired by Kaplan–Meier Plotter using a log-rank test, and *p* < 0.05 was considered significant. * = significant value.

The peripheral expression of *OXTR* (Figure 3A) was significantly low (*p* = 0.0387) in the metastatic group of patients as compared to the non-metastatic patients (Figure 3B). A near-significant difference was found in terms of tumor grades (Figure 3C). Similar to *5-HTT* and *NR3C1*, the expression of *OXTR* is also related to the clinical outcomes of breast cancer. Kaplan–Meier analysis showed a high relapse-free survival probability in the patients with high expressions of *OXTR* (Figure 3D), suggesting its downregulation is associated with poor disease outcomes.

#### 3.2.4. FKBP Prolyl Isomerase 5 (*FKBP5/Ptg-10*) in Breast Cancer

**Figure 4:**
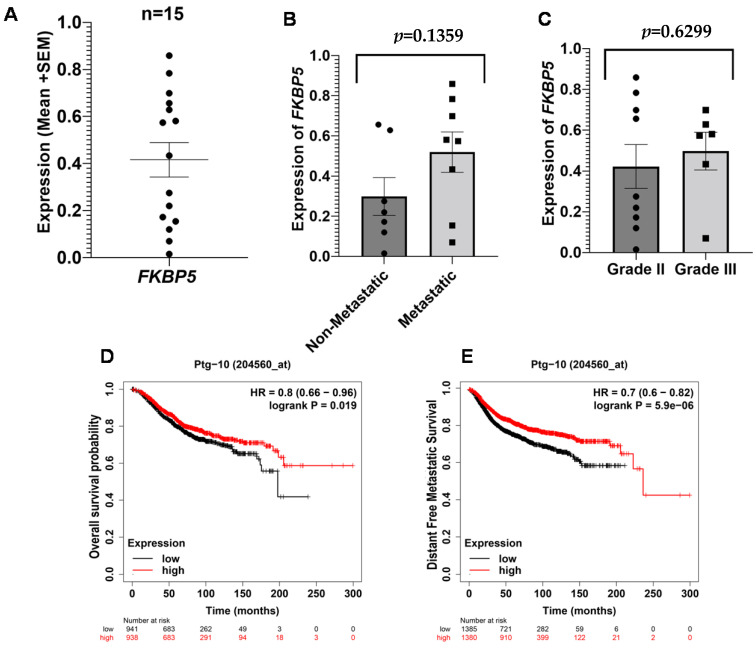
Expression of *FKBP5* and survival outcomes in breast cancer patients. Peripheral expression of *FKBP5* across all breast cancer patients (**A**). Difference in the expression of *FKBP5* between non-metastatic and metastatic patients (**B**). Difference in the expression of FKBP5 between grade II and grade III (**C**). Kaplan–Meier Survival plots for OS (**D**) and DFMS (**E**). The results were acquired by Kaplan–Meier Plotter using the Kaplan–Meier method with a log-rank test, and *p*-values less than 0.05 were considered statistically significant.

The role of *FKBP5/Ptg-10* in stress-related conditions like post-traumatic stress disorder and depression was established in previous studies [23,24]. We examined the mRNA expression of *FKBP5* in our subjects (Figure 4A) and found a relatively higher expression of *FKBP5* in metastatic patients and in patients with advanced-grade tumors (Figure 4B,C), but differences in the values were not statistically significant. Survival curves showed that patients with high mRNA levels of *FKBP5* have high overall and distant-free metastatic survival probability (Figure 4D,E), indicating a positive prognosis in breast cancer morbidity.

## 4. Discussion

The main findings of our study are that the stress-associated genes are differentially expressed in metastatic patients compared to the non-metastatic controls and between grade II and III patient samples. The expression of *OXTR* (*p* = 0.03) and *5-HTT* (*p* = 0.02) was significantly reduced in metastatic patients, but no changes in gene expression were found between the grade II and grade III patients. However, *NR3C1* (*p* = 0.03) was downregulated in the grade III patients compared to the grade II patients, but no differences were observed in *NR3C1* expression between the metastatic and non-metastatic patients. Moreover, we also found a high expression of *NR3C1* (*p* = 0.03) in estrogen receptor-positive patients. Based on Kaplan–Meier analysis, we found that the low expression of these genes is associated with reduced survival probability in breast cancer patients. These results underscore the prognostic importance of stress-associated genes in the metastasis of breast cancer in human patients. Prior to studying the expression profiles, the stress evaluation of cancer patients should be performed to evaluate the presence of cancer-related stressors in our disease population.

There is evidence that *NR3C1, 5-HTT*, and *OXTR* are downregulated in stress-associated conditions [25,26,27]. The silencing of *5-HTT* resulted in enhanced anxiety levels in mice, and the effects were reversed when treated with the antagonist receptors [28]. Additionally, the modulation of the variable number of tandem repeat (VNTP) domain of the *5-HTT* gene was regulated by CTCF [29], a multifunctional transcription factor that was previously found to regulate the tumor-suppressor activity of *HOXA10* in breast cancer [30].

The role of *OXTR* is also established in regulating the stress response [31]. Even though our results showed a low expression of *OXTR* in high metastatic patients, the previous studies explored that its overexpression is associated with increased cell migration and the proliferation of cancer cells via the pSTAT5 pathway [32]. Particularly, the overexpression of this gene reduces the survival chances of patients with triple-negative breast cancer [33]. The role of *OXTR* in tumorigenesis should be assessed extensively in breast cancer to identify its concrete role in all breast cancer subtypes.

*NR3C1* is downregulated in the hippocampus of mouse brain in acute stressful conditions. It is regulated by DNA methyltransferase alpha (*DNMT3a*) and miRNA-124a [34]. *DNMT3a* is significantly upregulated in breast tumorigenesis [35,36,37,38]. It suggests that a global increase in *DNMT3a* in a highly stressed breast cancer patient might result in the silencing or downregulation of *NR3C1* and interfere with the regulation of the stress response, mediated by *NR3C1*. However, the overexpression of miRNA-124a was significantly associated with anti-tumor properties [39,40]. Specific studies should be conducted to explore this potential pathway in the signaling of stress-associated cancer progression.

Even though we did not find any significant difference in the FKBP5 mRNA levels in different groups, the survival plots suggest high overall and distant free metastatic probability in patients with a high expression of *FKBP5* (Figure 4D,E). *FKBP5* is implicated in regulating cancer-related signaling pathways that can lead to chemoresistance in cancer patients [41,42,43]. A study published in 2015 claimed the inhibitory and apoptotic effects of *FKBP5* on the proliferation of glioma cells [44], suggesting its alternative roles in different cancers.

Survival analysis using the KM plotter showed that the low expression of all these genes is associated with less survival probability in breast cancer patients (Table 4). It can be suggested that the low expression of these genes in metastatic breast cancer patients may be due to their emotionally unstable condition, or perhaps their downregulation induces a metastatic switch and enhances cancer progression.

Stress-related triggers of metastasis are of great interest to researchers and scientists working in psycho-oncology. However, underlying molecular mechanisms should also be studied along with the social and behavioral patterns of stress in cancer. By studying the mRNA expression profiles of stress-associated genes in breast cancer patients based on their histological grade and metastatic status, we were able to identify to what extent these genes express differently in clinically diverse breast cancer patients. The study validates the potential involvement of stress-related mediators in cancer progression and provides a framework for future researchers to identify signaling pathways to fill the gaps between stress and cancer pathophysiology.

## 5. Conclusions

The current study found the dynamic expression of these stress-associated genes in a heterogenous group of breast cancer patients, suggesting their possible impact on cancer progression and prognosis. These genes cannot only act as biomarkers of stress and depression in cancer patients but also as new therapeutic targets in the field of pharmacology. The identification of signaling cascades connecting emotional stress to cancer pathophysiology is significant for unconventional diagnostic approaches and stress management. Additionally, the therapeutic potential of these genes should be assessed in all molecular subtypes to provide personalized options for treating breast cancer.

## Figures and Tables

**Table 1:** Clinical and demographical profiles of breast cancer patients.

Clinical Characteristics	Patients (n)
**Age**	
Less Than 35	5
35–45	10
45–55	12
Above 55	5
**Family History**	
Present	8
Not present	24
**Mastectomy**	
Yes	21
No	11
**Tumor Grade**	
II	20
III	12
**Metastasis**	
Yes	18
No	14
**ER status**	
Positive	13
Negative	15
Unknown	4
**PR status**	
Positive	11
Negative	21
**HER2 neu status**	
Positive	12
Negative	20

**Table 2:** List of oligonucleotide sequences.

Gene	Forward Primer	Reverse Primer	Product Size
**NR3C1**	5′GCTGGAATGAACCTGGAAG3′	5′ACAGTGACACCAGGGTAGGG3′	157 bp
**FKBP5**	5′ACTGTTGCTGAGCAGGGA3′	5′CCATGCCTTGATGACTTGGC3′	221 bp
**5-HTT**	5′ATCATCCTTTCTGTCCTGCTGG3′	5′CCGGACCAAGAGAGAAGAAGAT3′	151 bp
**OXTR**	5′ CCTTCATCGTGTGCTGGAC 3′	5′ CGAGTTCGTGGAAGAGGTG3′	174 bp
**ACTIN**	5′ CCATGTACGTTGCTATCCAG 3′	5′ CCATCTCTTGCTCGAAGTC3′	295 bp

**Table 3:** Subgroup analysis of QSC-R10 among different variables in breast cancer patients (n = 32).

Variables (n)	Mean Scores	*p*-Value
**Age**		
<35 (n = 5)	3.30 ± 0.20 ^a,b^	0.0365 *
35–45 (n = 10)	3.59 ± 0.37 ^a^	
45–55 (n = 12)	3.15 ± 0.34 ^b^	
>55 (n = 5)	3.17 ± 0.31 ^b^	
**Mastectomy**		
Yes (n = 21)	3.45 ± 0.35 ^a^	0.03 *
No (n = 11)	3.05 ± 0.38 ^b^	
**Illness duration**		
<6 months (n = 9)	3.58 ± 0.29 ^a^	0.0011 **
6 months–2 years (n = 6)	3.18 ± 0.14 ^b,c^	
2 years–3.5 years (n = 9)	3.41 ± 0.42 ^a,b^	
>3.5 years (n = 8)	2.98 ± 0.19 ^c^	
**Metastasis**		
Yes (n = 18)	3.38 ± 0.36	0.14 ^NS^
No (n = 14)	3.22 ± 0.41	

* Significant (*p* < 0.05); ** Highly significant (*p* < 0.01); NS = Non-significant (*p* ≥ 0.05); Mean scores with different alphabetical indicators in a parameter are statistically significant.

**Table 4:** Kaplan–Meier survival analysis in breast cancer.

Gene Name	AFFY METRIX ID	OS *p*	HR	RFS *p*	HR	DFMS *p*	HR	PPS *p*	HR
*NR3C1*	201865_x_at	0.0003 *	0.72	4.2 × 10^−9^ *	0.74	0.4	0.92	0.13	0.8
*5-HTT*	207519_at	0.03 *	0.82	4.3 × 10^−14^ *	0.6	0.1	0.85	0.0011 *	0.72
*OXTR*	206825_at	0.09	0.85	0.0009 *	0.84	0.5	0.91	0.6	0.9
*FKBP5*	204560_at	0.01 *	0.78	0.32	1.06	5.9 × 10^−6^ *	0.78	0.4	0.9

OS = Overall survival, RFS = relapse-free survival, DFMS = Distant free metastatic survival, PPS = Post progression survival; HR = Hazard ratio; Significant data are highlighted by *.

## Data Availability

All the data generated in this study have been included in the manuscript.

## Supplementary Materials

The following supporting information can be downloaded at: https://www.mdpi.com/article/10.3390/ijms232214097/s1, Figure S1: Questionnaire on Distress in Cancer Patients–short form (QSC-R10).
