# Supplementary material for: Peripheral mRNA Expression and Prognostic Significance of Emotional Stress Biomarkers in Metastatic Breast Cancer Patients
Source: Int J Mol Sci. 2022 Nov 15;23(22):14097. doi: 10.3390/ijms232214097 (PMC9694977; doi:10.3390/ijms232214097)
Supplement: Supplementary file 1 [file ijms-23-14097-s001.zip › ijms-1954545-Supplementary.pdf]

Here is a list of **potentially stressful situations** which might apply in your daily life. For each situation, please decide whether the situation applies to you. If so, please indicate how serious the problem is for you by checking the box on the five-point scale "a slight problem" to "a very serious problem". If not, then check the box "not applicable".

QSC - R10

	Not applicable	Applies to me and is a slight problem				a very serious problem
1. I feel tired and weak.	0	1	2	3	4	5
2. I am in pain.	☐	☐	☐	☐	☐	☐
3. I feel physically imperfect.	☐	☐	☐	☐	☐	☐
4. I have too few opportunities to speak with a professional about my psychological distress.	☐	☐	☐	☐	☐	☐
5. I am afraid that my disease will spread/recur.	☐	☐	☐	☐	☐	☐
6. It is difficult for my partner to empathize with my situation.	☐	☐	☐	☐	☐	☐
7. My sleep is disturbed.	☐	☐	☐	☐	☐	☐
8. It is harder for me to take part in recreation activities (e.g. sports) now than it was before I became ill.	☐	☐	☐	☐	☐	☐
9. I do not feel well informed about my disease/treatment.	☐	☐	☐	☐	☐	☐
10. I feel tense and/or nervous.	☐	☐	☐	☐	☐	☐

© Herschbach 2008

Figure S1: Questionnaire on Distress in Cancer Patients–short form (QSC-R10).
